# EHMTI-0025. Clinical manifestations of subarachnoid hemorrhage from gnathostoma spinigerum in srinagarind hospital

**DOI:** 10.1186/1129-2377-15-S1-D55

**Published:** 2014-09-18

**Authors:** K Sawanyawisuth, W Waranon, K Pongtipakorn, K Kongbunkiat, P Limpawattana, V Senthong, J Chindaprasirt, V Chotmongkol, J Kanpittaya, PM Intapan, W Maleewong, A Kitkhuandee

**Affiliations:** 1 Medicine, Khon Kaen University, Khon Kaen, Thailand; 2 Radiology, Khon Kaen University, Khon Kaen, Thailand; 3 Parasitology, Khon Kaen University, Khon Kaen, Thailand; 4 Surgery, Khon Kaen University, Khon Kaen, Thailand

## Introduction

Subarachnoid hemorrhage (SAH) is a serious neurological condition. Common cause of SAH is vascular origin. Gnathostomiasis is also a common disease in Thailand and may cause SAH.

## Aim

This study aimed to find clinical differences between SAH caused by both causes.

## Methods

This was a retrospective study and collected data from medical charts of patients diagnosed as SAH at Srinagarind Hospital during 2009 and 2011. SAH caused by vascular causes diagnosed by cerebral angiogram, while cerebral gnathostomiasis diagnosed by negative cerebral angiogram with positive gnathostoma antibody. Clinical features between both groups were compared by descriptive statistics.

## Results

There were 18 patients in vascular group and 10 patients in gnathostomiasis group. Most variables between both groups were comparable except cerebrospinal fluid glucose/plasma glucose. This ratio in gnathostomiasis group was significantly higher than vascular group (80% vs 16.67%, respectively).

## Conclusion

Cerebrospinal fluid glucose/plasma glucose ratio was significantly higher in SAH patients caused by gnathostomiasis than vascular group.

No conflict of interest.

